# Metastatic Transitional Cell Carcinoma of the Bladder to the Testis: A Case Report

**DOI:** 10.1155/2012/486245

**Published:** 2012-10-22

**Authors:** Gregory N. Kozak, Nicholas C. Field

**Affiliations:** Southern Alberta Institute of Urology, University of Calgary, Suite 6645, 7707-14 Street SW, Calgary, AB, Canada T2V 1P9

## Abstract

An 84-year-old gentleman presented with onset of gross hematuria in September 2010. Follow-up investigations revealed T1 superficially invasive, poorly differentiated, papillary urothelial carcinoma. He subsequently had GreenLight laser for BPH and bladder neck contracture on two occasions. He developed a right hydrocele 16 months after initial presentation and during his hydrocelectomy, a rock-hard right epididymis and testicle were discovered. Pathology revealed metastatic urothelial carcinoma replacing nearly the entire testis with lymphovascular invasion.

## 1. Introduction

Bladder cancer is the 5th most commonly diagnosed cancer in the USA and the 2nd most common genitourinary cancer after prostate cancer [1, 2]. In Western countries, the major risk factors are environmental exposure such as smoking and occupational exposure to chemicals used in the dye, rubber, leather, and painting industries [3]. The most common symptoms of bladder cancer are painless hematuria, urgency, frequency, and irritation associated with voiding [3].

Histologically, bladder cancer is categorized into transitional cell carcinoma (90%), squamous cell carcinoma (5%), and adenocarcinoma (<2%) [2]. Non-invasive TCC is divided into papillary and flat. Noninvasive papillary TCC is subdivided into papilloma, papillary transitional cell neoplasm of low malignant potential, low grade carcinoma, and high grade carcinoma [2].

## 2. Case Presentation

An 84 year old gentleman presented to the Emergency Department with onset of gross hematuria in September 2010. Subsequent pelvic ultrasound revealed a 1.2 × 1.6 × 1.5 cm lesion on the anterior wall of the urinary bladder projecting into the lumen. Cystoscopy showed a small bladder tumor below the bladder neck and a larger tumor to the left of the original tumor. TURBT pathology indicated a T1 superficially invasive poorly differentiated papillary urothelial carcinoma, focally invasive into the lamina propria, but not the muscularis propria. No CIS was noted. TURP pathology indicated inflammatory tissue consistent with previous infection of the prostate.

Follow-up cystoscopy in January 2011, showed no evidence of recurrent tumor and the patient was scheduled to start BCG therapy. On repeat cystoscopy in May 2011, a bladder neck contracture was noted. GreenLight Laser for BPH and bladder neck contracture was performed in June 2011. During follow up cystoscopy in November 2011, an additional bladder neck contracture was identified and this was subsequently treated with GreenLight laser in December 2011. Prior to the second GreenLight laser treatment, an ultrasound for scrotal enlargement revealed bilateral hydroceles, right greater than the left, and heterogeneity of the right testicle. Follow-up ultrasound 3 months later revealed an increase in size of the right hydrocele, with the testicles appearing symmetric and normal other than a suspected cyst within the left testicle.

Six sessions of BCG therapy were completed by March 2012 and follow-up cystoscopy in April revealed a normal-appearing bladder with no recurrent tumor and a minimal bladder neck contracture.

In May 2012, a scheduled right hydrocelectomy was performed, however, following drainage it was discovered that the right epididymis and testicle had the consistency of a hard rock. As a result of this unexpected finding, a right radical orchiectomy was performed. Pathology of the right testicle and spermatic cord revealed metastatic urothelial carcinoma replacing nearly the entire testis with lymphovascular invasion. This was diffusely positive for CK7, focally strongly positive for CK5/6 and CK20, and focally strongly positive for p63 with nuclear staining. One paratesticular lymph node was positive for metastatic urothelial carcinoma. The spermatic cord and resection margins were negative for urothelial carcinoma. No other sites of metastatic spread were noted.

## 3. Discussion

The incidence of transitional cell carcinoma metastasizing to the testis is exceptionally rare. A MEDline search from 1966 to present identified only 8 previous occurrences. The most common sites for metastasis of primary transitional cell carcinoma is to the lymph nodes (78%), liver (38%), lung (36%), bone (27%), adrenal glands (21%), and intestines (13%) [4]. Typically, those who present with metastatic disease have primary tumors of high grade disease when the diagnosis is made [4]. Known primary tumors that metastasize to the testis include prostate, lungs, GI tract, melanoma, and the kidney [5].

Of particular interest in this case is whether the previous transurethral resections could have provided a seeding mechanism that encouraged metastatis growth. While metastatic of TCC to the testis is extremely rare, this case illustrates that metastatic neoplasm to uncommon sites should be considered in the differential for patients presenting with TCC of the bladder.
